# Three-Dimensional-Printed Guiding Template for Unicompartmental Knee Arthroplasty

**DOI:** 10.1155/2020/7019794

**Published:** 2020-12-19

**Authors:** Fei Gu, Liangliang Li, Huikang Zhang, Xuxiang Li, Chen Ling, Liming Wang, Qingqiang Yao

**Affiliations:** ^1^Department of Orthopaedics, Nanjing First Hospital, Nanjing Medical University, No. 68, Changle Rd, Qinhuai District, Nanjing 210006, China; ^2^Institute of Digital Medicine, Nanjing Medical University, No. 68, Changle Rd, Qinhuai District, Nanjing 210006, China; ^3^The Second People's Hospital of Lianyungang, No. 41, Hailian East Rd, Haizhou District, Lianyungang 222000, China; ^4^Department of Orthopedics, The Affiliated Jiangning Hospital with Nanjing Medical University, Nanjing 211100, China; ^5^Nanjing Clinical Nuclear Medicine Center, Nanjing First Hospital, Nanjing Medical University, No. 68, Changle Rd, Qinhuai District, Nanjing 210006, China

## Abstract

**Background:**

For unicompartmental knee arthroplasty (UKA), accurate alignment of the limb is crucial. This study is aimed at investigating the efficacy and safety of a three-dimensional printed patient-customized guiding template (3DGT) for UKA.

**Methods:**

A total of 22 patients receiving UKA were randomly divided into the 3DGT-UKA group (*n* = 11) and traditional UKA group (T-UKA group; *n* = 11). In the 3DGT-UKA group, the line and angle of osteotomy were decided on a 3D image of the limb reconstructed from imaging data; a guiding template was then designed and printed out. The patients in the T-UKA group underwent conventional UKA. Prosthesis size, operation time, postoperative drainage, hip–knee angle (HKA), pain, and Hospital for Special Surgery (HSS) scores were recorded at day 1, week 1, month 1, and month 3 after surgery.

**Results:**

There was no significant difference in the size of prostheses between the preoperatively designed and actually used in the 3DGT-UKA group (*p* > 0.05). HKA was comparable in 3DGT-UKA and T-UKA patients. Operation time was shorter (53.6 ± 6.4 minutes vs. 75.8 ± 7.1 minutes) and wound drainage was less (93.2 ± 3.9 mL vs. 85.2 ± 3.0 mL) in 3DGT-UKA than in T-UKA (*p* < 0.05). Hospital stay was shorter in the 3DGT-UKA group. The 3DGT-UKA group had a lower VAS score on day 1, week 1, and month 1 and a higher HSS score on week 1 and month 1 after surgery. No varus/valgus deformity or prosthesis loosening was observed in either group at the final follow-up.

**Conclusion:**

The 3D-printed patient-customized guiding template may help decrease operation time, decrease blood loss, and improve short-term clinical outcomes in patients undergoing UKA surgery.

## 1. Introduction

The prevalence of osteoarthritis (OA) of the knee has increased rapidly with the aging of the population, and it has become a major cause of pain and disability. For medial compartment knee osteoarthritis (MOA), unicompartmental knee arthroplasty (UKA) has several advantages over total knee arthroplasty (TKA), including the preservation of almost all functions of the knee joint, lower postoperative complications rate, and quicker postoperative functional recovery. However, for successful UKA, accurate alignment of the limb is crucial; even a minimal shift may decrease the lifespan of the prosthesis and lead to a need for revision surgery [1, 2]. Currently, the balance of soft tissues, the selection of prosthesis size, the angle of osteotomy, and related lower limb alignment are mainly decided by visual assessment of the surgeon. Therefore, the experience of the surgeon is one of the key factors for the reasonable performance of UKA. However, individual variations between patients and the lack of familiarity of the surgeon with the medical apparatuses might influence the final surgical effect [3].

In recent years, there has been much research on the use of three-dimensional (3D) digital image design and 3D-printing technology to build personalized guiding templates for knee surgery. A 3D-printed individualized guiding template based on CT-MRI fusion data could greatly improve the accuracy of estimation of the angle, the amount of osteotomy, the sizes of the tibial platform, and the femoral condyle prosthesis [4–7]. The aim of this randomized clinical trial was to investigate the efficacy and safety of the use of 3D-printed individual patient-customized guiding templates for assisting UKA.

## 2. Materials and Methods

### 2.1. Patients

**Figure 1:**
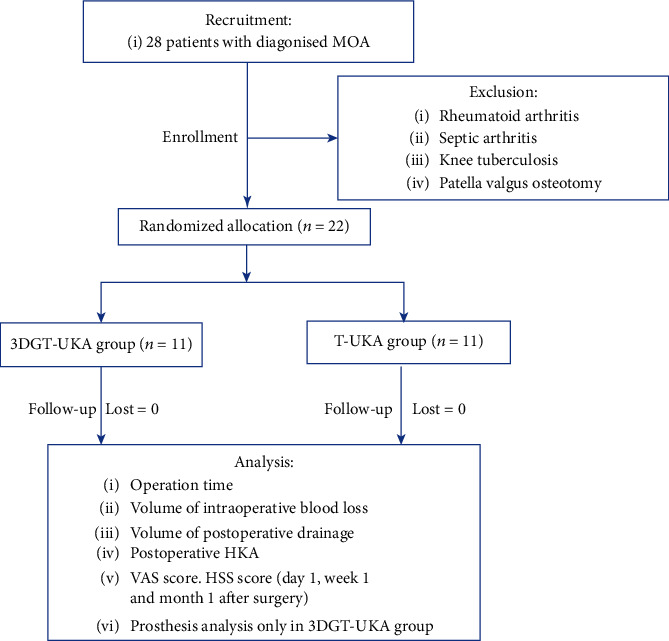
Consort diagram.

This randomized clinical trial was performed at the Department of Orthopedics, Nanjing First Hospital, affiliated to Nanjing Medical University. MOA patients requiring UKA surgery during the period of January 2017 to December 2017 were enrolled in this study. Patients were eligible for inclusion if they had (1) a diagnosis of MOA confirmed by X-ray and MRI, (2) intact anterior cruciate ligament, and (3) knee varus deformity that could be corrected manually. Patients with a history of rheumatoid arthritis, septic arthritis, knee tuberculosis, and patella valgus osteotomy were excluded. A total of 22 patients who met these criteria were enrolled and randomly assigned to receive either 3D-printed patient-customized guiding template-assisted UKA (3DGT-UKA group) or traditional UKA (T-UKA group) (Figure 1).

This study was approved by the Ethics Committee of Nanjing Hospital affiliated to Nanjing Medical University. All patients gave written informed consent for participation in the study.

### 2.2. Preoperative Preparation

Preoperatively, all patients underwent electrocardiography, routine blood examination, and vascular ultrasonography of the lower limb. Patients with comorbidities received appropriate specialist consultations to rule out contraindications to surgery.

### 2.3. Design and Production of 3D-Printed Personalized Guiding Template for UKA Osteotomy

**Figure 2:**
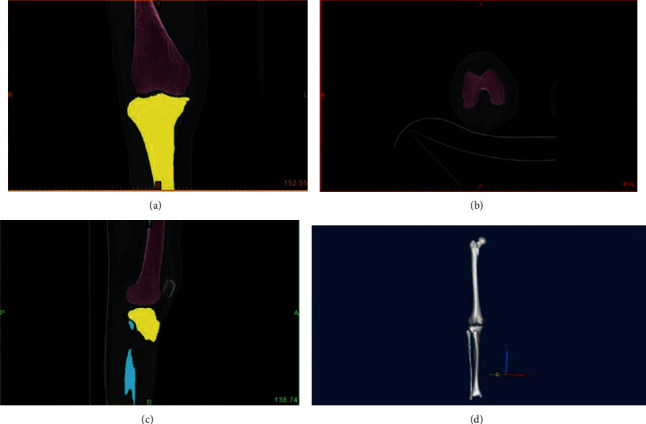
(a) Front view CT image of the knee joint. (b) Top view CT image of the knee joint. (c) Side view CT image of the knee joint. (d) Reconstructed three-dimensional model of a lower limb in full length.

**Figure 3:**
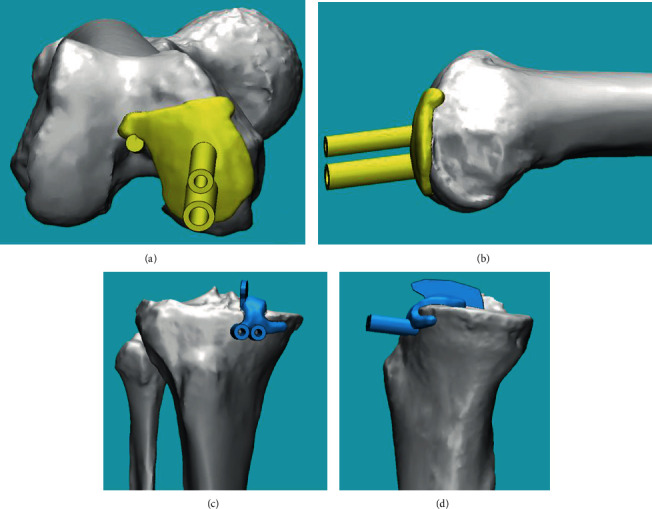
(a) Front view of the femoral 3D-printed guiding template. (b) Side view of the femoral 3D-printed guiding template. (c) Front view of the tibial 3D-printed guiding template. (d) Side view of the tibial 3D-printed guiding template.

Patients in the 3DGT-UKA group first underwent a double-source 64-slice spiral CT (Siemens Sensation, Germany) full-length scan of the lower limbs and MRI scans of the knee joints. The data were imported into a workstation. CT data were processed using the Mimics 17.0 software (Materialise Ltd., Leuven, Belgium) to reconstruct a 3D model of the full length of the lower limbs. The MRI data were processed to obtain a 3D model of the knee joint. The two models were then merged to obtain a 3D model of the lower limbs with the cartilage lining of the knee joints also displayed. The angle between the hip and knee (HKA) and the mechanical axis of the femur and the tibia were measured using a computer-aided design (CAD) software (Medivi, Changzhou, China). The angle and amount of osteotomy were accurately calculated on the reconstructed 3D model, and the posterior oblique angle and amount of osteotomy necessary at the tibial plateau were determined. Finally, a 3D-printed customized guiding template was created for each patient. The template was sterilized with plasma before use (Figures 2 and 3).

### 2.4. Surgical Methods

**Figure 4:**
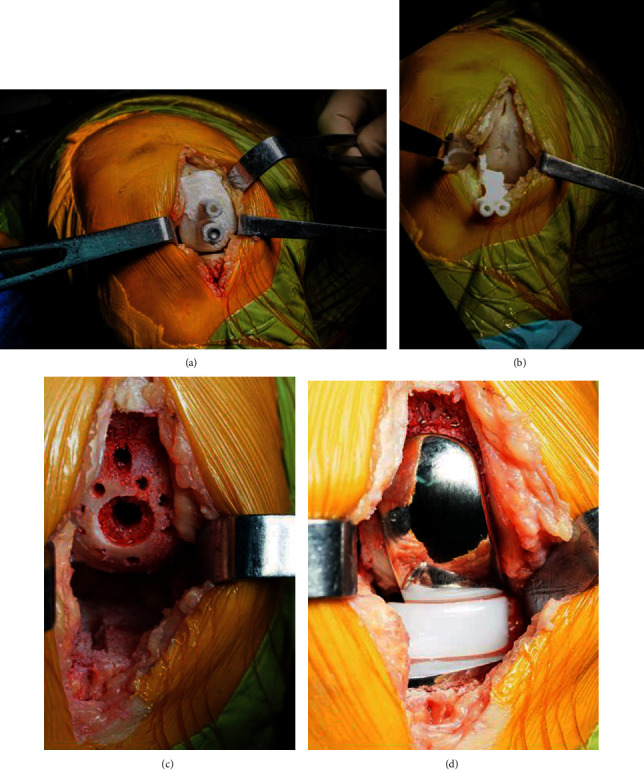
(a) Intraoperative placement of the femoral condyle 3D-printed guiding template. (b) Intraoperative placement of the tibial plateau 3D-printed guiding template. (c) Images of the femoral and tibial osteotomy. (d) Images of the installed prosthesis.

The 3rd generation Oxford prostheses for UKA were used for all patients. All surgeries were performed by the same surgical team. Surgery was performed under general anesthesia. Patients in the 3DGT-UKA group were in the supine position, and a tourniquet was placed at the base of the affected limb. The hip was flexed 30° and mildly abducted, with the calf hanging naturally down. Free movement of the knee joint for at least 135° was ensured. With the knee flexed at 90°, a 6-cm incision was made on the medial side of the knee joint, extending from the distal end of the joint line at the medial edge of the tibia up to the medial edge of the femoral muscle. The distal femoral fat pad was removed to expose the distal femur and the superior tibia. The femoral condyle, intercondylar fossa, tibial plateau epiphysis, and the soft tissue attached to the anterior side of the platform were removed. Then, the tibial 3D-printed patient-customized guiding template was carefully and closely applied on the anterior aspect of the tibial plateau, and with its guidance holes were drilled into the proximal end of the tibia. The positioning nails were inserted, and the 3D-printed patient-customized guiding template was removed. Next, the manufacturer-made matching osteotomy template (Zimmer Ltd., USA) was then positioned, and osteotomy of the tibial plateau was carried out. After the osteotomy, the dimensions of the cut tibial plateau and prosthesis were measured. The distal femur and intercondylar fossa were then exposed. The distal femoral 3D-printed guiding template was placed in position, and the drilling was carried out. The distal femoral template was removed, and the manufacturer-made posterior condylar template (Zimmer Ltd., USA) was placed for posterior condylar osteotomy. The bone bung was inserted into the distal femur for grinding. Finally, a single condylar prosthesis of the appropriate preoperatively determined size was then installed (Figure 4).

In the T-UKA group, the conventional single condylar replacement method was used. Tibial osteotomy aimed at making a downward 7° of posterior tilt in the tibial plateau. The thickness of the tibial osteotomy was 2-3 mm less than the depth of the deepest part of the tibial wear. Osteotomy of the medial femoral condyle was performed by intramedullary positioning. After drilling the anterior angle of the anterior intercondylar fossa, the rods for intramedullary positioning were inserted. The femoral drill guide was placed, ensuring that the handle was parallel with the long axis of the tibia. The kneading surface was close to each other, and the relevant angle was adjusted. The positioning holes (diameter of 4 mm and 6 mm) were drilled and according to the position of them; the posterior femoral condyle was cut and ground. Prostheses of appropriate sizes were then installed into the tibia and femur.

**Figure 5:**
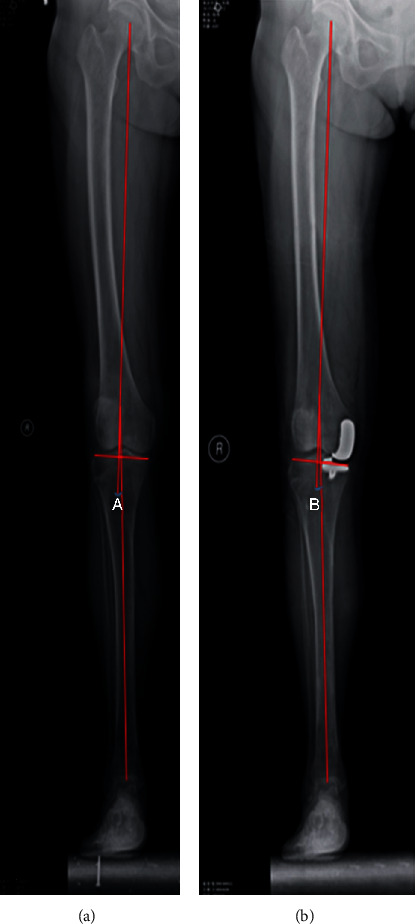
(a) Preoperative lower limb line (angle between the hip and knee (A) = 3.5°). (b) Postoperative lower limb line (angle between the hip and knee (B) = 2.4°).

Drainage tubes were placed in both groups after surgery [8–10]. All patients routinely received antibiotics and analgesics, as well as low-molecular-weight heparin as an anticoagulant therapy. Passive exercises were started 24 hours after surgery. The drainage tube was removed 48 hours after surgery. Patients were encouraged to walk with the help of a walking assistance device and to perform stretching and contracting exercise of quadriceps. A full-length radiograph of the affected lower limb and a positive lateral radiograph of the knee joint were examined at 2 days postoperation, and the HKA deviation was obtained (Figure 5). Sutures were removed 2 weeks after surgery. The VAS scores on day 1, week 1, month 1, and month 3 after surgery, and the Hospital for Special Surgery (HSS) scores on week 1, month 1, and month 3 after surgery, were recorded.

### 2.5. Postoperative Evaluation

An outpatient follow-up for three months was conducted to evaluate the recovery of the patients. And all patients involved in this study completed the postoperative follow-up. The sizes of the preoperatively designed theoretical prosthesis and that of the actual prosthesis used during the operation in the 3DGT-UKA group were compared. The operation time, intraoperative blood loss, postoperative drainage, and postoperative HKA were compared between the two groups. VAS score on day 1, week 1, month 1, and month 3 after surgery and HSS score on week 1, month 1, and month 3 were also compared between the two groups. Because the operation time of patients in both groups was within the set time (90 minutes) of a lower extremity tourniquet, the volume of postoperative drainage was recorded and calculated as the volume of blood loss in the current study.

### 2.6. Statistical Analysis

Statistical analysis was performed using the SPSS 22.0 statistical software (IBM Corp., Armonk, NY, USA). The rank-sum test was performed for ranked data in the two groups. The *t*-test was used for the analysis measurement data and the chi-squared test for enumeration data. Statistical significance was at *p* ≤ 0.05.

## 3. Results

A total of 22 patients (8 males, 14 females) with mean age 60.8 ± 4.8 years (age range, 52-67 years) were enrolled and randomized to receive either 3DGT-UKA (*n* = 11) or T-UKA (*n* = 11). The gender ratio, mean age, mean height, preoperative HSS score, and prevalence of comorbidities were comparable in the two groups (Table 1). In the 3DGT-UKA group, no significant difference was seen between the sizes of actual prosthesis used and preoperative theoretical prosthesis model (*p* > 0.05; Table 2). There was no significant difference between the two groups in postoperative HKA deviation (*p* > 0.05; Table 3). Operation time was significantly shorter in the 3DGT-UKA group than in the T-UKA group (53.6 ± 6.4 minutes vs. 75.8 ± 7.1 minutes; *p* < 0.05). The volume of postoperative drainage was significantly lower in the 3DGT-UKA group than in the T-UKA group (67.5 ± 3.9 mL vs. 93.2 ± 3.0 mL; *p* < 0.05). The mean hospitalization time was longer in the T-UKA group than in the 3DGT-UKA group. The VAS scores on day 1, week 1, and month 1 after surgery were significantly lower in the 3DGT-UKA group than in the T-UKA group (*p* < 0.05). And the HSS scores on week 1 and month 1 after surgery were higher in the 3DGT-UKA group than in the T-UKA group (*p* < 0.05). The HSS scores on month 3 after surgery were comparable between the two groups (*p* > 0.05; Tables 4 and 5).

## 4. Discussion

In theory, for OA of the knee with only medial compartment lesions, UKA may be a reasonable candidate by the preservation of the anterior-posterior cruciate ligaments. This means that most functions of the knee joint may be retained after UKA surgery. Previous studies proved that UKA showed significant advantages in symptom relief, knee function recovery, and postoperative complications, compared to TKA [8–11]. However, the long-term studies showed that the survival of prostheses in UKA was significantly lower than TKA, which may be caused by the inadequate surgical exposure due to the small incision used in UKA, large errors in intraoperative extracorporeal positioning of the lower extremity force line, and intramedullary positioning of the femoral condyle [12]. It has been confirmed that a slight error in the alignment of the lower limb line and a lower extremity varus increases the need for future revision surgery in patients receiving UKA. Meanwhile, postoperative valgus can significantly increase lateral compartment stress and lead to accelerated cartilage degeneration in the lateral compartment of the knee joint [13]. Therefore, in UKA patients, detailed preoperative planning and precise positioning of the intraoperative prosthesis is crucial.

Routine analysis of radiography, CT, and MRI images of the knee joint is difficult to accurately obtain the 3D structure of the lower extremities, and it is also difficult to quantitatively show the cartilage wear of the medial compartment. It looks like the measurement error cannot be avoided during the operation because the positioning of the tibial plateau in T-UKA patients depends on the extramedullary positioning rods, which is subjective. Furthermore, deviations in the intramedullary positioning of the distal femoral condyle and any other errors in the direction of the positioning rod may lead to impingement or unsatisfactory meniscus trajectory, as well as prolonged operation time, increased blood loss, and the risk of intraoperative fat embolism. Ma et al. studied the positional differences of unicompartmental prostheses caused by the deviation of the intramedullary positioning entrance points in the femurs of 20 corpses and concluded that the intramedullary positioning rods could not accurately indicate the actual anatomical axis of the femur, which usually leads to a deviation with 3.2° of deflection and 2.5° of valgus [14]. Baldini et al. found that 10%-20% of patients who received T-UKA had a significant deviation of the femoral force line on the prosthetic side from the normal [15]. Therefore, we felt that a 3D-printed individualized guiding template based on quantitative anatomical data and accurate preoperative CAD measurement results would be helpful in the selection of the appropriate osteotomy angle and amount. In addition, there is no need for opening of the femoral bone marrow during femoral osteotomy with 3DGT-UKA, which helps reduce the surgical exposure time, postoperative blood loss, and amount of intraoperative instrumentation.

A 3D-printed guiding template based on CT data alone is difficult to achieve good intraoperative fitting of the guiding template due to it does not take into consideration the cartilage tissue influence, which has been shown to be responsible for the decrease in the accuracy of the 3D-printed guiding template [16]. Therefore, in this present study, the CT and MRI data were merged to reconstruct the 3D anatomy of the knee joint, which allowed accurate calculation of the lower limb force line, angle, and osteotomy amount. This method can help us design an accurate 3D-printed patient-customized guiding template during UKA surgery. Due to the refined and minimally invasive design of the third-generation Oxford knee prosthesis for UKA, the flexible meniscus pad also reduces the risk of impingement and rotation of the prosthesis. Therefore, the design of the 3D-printed guiding template in this study was based on the third-generation Oxford UKA tool kit. We found there was no significant statistical difference in dimensions between the preoperatively designed theoretical prosthesis and the actual prosthesis used in the 3DGT-UKA group. There was also no significant difference in HKA between the 3DGT-UKA and T-UKA groups, indicating the accuracy of the 3DGT-UKA surgery [17, 18].

Compared to the 3DGT-UKA group, which prosthesis selection is based on accurate preoperative measurements of the reconstructed 3D knee joint, leading to less errors and rapid surgery completion [19, 20], the T-UKA group select the prosthesis model based on preoperative radiographic measurements, which is subjective and may cause large errors. Therefore, repeated measurements are necessary during surgery to select the appropriate size. In addition, the 3D-printed guiding template easily and accurately locates the surgical osteotomy tool without slotting, which also contributes to the decrease in operation time and postoperative drainage volume. Also, we found the 3D-printed guiding template can help to reduce surgical trauma during UKA surgery and the duration of tourniquet application, which is probably related to the lower VAS score on day 1 and week 1 and higher HSS score on week 1 and month 1 after surgery in the 3DGT-UKA group.

There are also some disadvantages with 3DGT-UKA. On the one hand, patients undergoing 3DGT-UKA need preoperative full-length CT and MRI scans, which increase medical costs and extra radiation exposure. On the other hand, specialized knowledge and special hardware and software are necessary for designing and printing an accurate 3D guiding template. All of these would increase the patient's economic burden. As for this study, we should also emphasize that it has some limitations. First, since the three-dimensional-printed guiding template for UKA is a new technique and the exclusion criteria are very strict, there was not much sample number. Second, we only collected the data of short-term outcomes; maybe we should focus on the midterm and long-term follow-up between these two groups in the further study.

To summarize, the 3D-printed guiding template can provide useful assistance for preoperative planning, intraoperative positioning, and osteotomy during UKA. It shortens the operation time, minimizes surgical trauma, and achieves better short-term clinical outcomes. In addition, the long-term follow-up outcomes need further research.

## Figures and Tables

**Table 1:** Demographic and clinical characteristics of patients in the 3DGT-UKA group and T-UKA group.

Group	Number	Gender		Age (yr, mean ± SD)	Height (cm, mean ± SD)	Difference of knee joints		Pre-operative HSS score (mean ± SD)	Alignment of lower limb, °^−^(mean ± SD)	Hypertension, *n* (%)	Diabetes, *n* (%)
		Male	Female			Left	Right				
3DGT-UKA	11	3	8	60.00 ± 4.31	159.55 ± 7.94	6	5	77.82 ± 2.44	7.23 ± 0.96	4 (36%)	2 (18%)
T-UKA	11	5	6	61.64 ± 5.33	161.64 ± 6.44	5	6	76.92 ± 2.47	7.19 ± 0.90	3 (27%)	2 (18%)
*t* value^1^				*−*0.791	−0.678			0.868	0.092		
*p* value^2^		0.659		0.438	0.505	1.000		0.395	0.928	1.000	1.000

^1^The *t* test was used for the comparison of age, height, preoperative HSS score, and alignment of preoperative lower limb. ^2^Fisher's exact test was used for comparison of gender ratio and surgery.

**Table 2:** Actual and theoretical values of surgical prosthesis models in the 3DGT-UKA group.

	3DGT-UKA femoral prosthesis model		3DGT-UKA tibial prosthesis model			
	M	S	C	B	A	AA
Theoretical value	1	10	0	4	5	2
Actual value	2	9	1	4	4	2
Test statistics	0.29	0.03	0.96	0	0.08	0
*t* value/*χ*^2^ value	0.588	0.867	0.328	1.000	0.779	1.000
*p* value	>0.05	>0.05	>0.05	>0.05	>0.05	>0.05

M, S, C, B, A, and AA represent the prosthesis model of the femoral and tibial.

**Table 3:** Comparison of HKA deviation in the 3DGT-UKA group and T-UKA group.

Group	Number	HKA deviation		
		> ±2°	±1° to ±2°	< ±1°
3DGT-UKA	11	3	5	3
T-UKA	11	4	4	3
*Z* value^1^		−0.28		
*p* value		0.78		

^1^Mann-Whitney *U* test.

**Table 4:** Comparison of surgical parameters and outcomes between the 3DGT-UKA group and the T-UKA group.

Group	Number	Operation time (min, mean ± SD)	Volume of postoperative drainage in 48 hours (mL, mean ± SD)	Postoperative HKA (°, mean ± SD)
3DGT-UKA	11	75.82 ± 6.91	85.18 ± 2.96	1.74 ± 0.78
T-UKA	11	67.64 ± 6.41	67.55 ± 3.86	1.81 ± 0.67
*t* value		2.879	12.031	*−*0.205
*p* value		0.009	0.000	0.840

**Table 5:** Comparison of postoperative VAS score and HSS score between the 3DGT-UKA group and the T-UKA group.

Group	Postoperative VAS score (mean ± SD)				Postoperative HSS score (mean ± SD)		
	Day 1	Week 1	Month 1	Month 3	Week 1	Month 1	Month 3
3DGT-UKA	4.64 ± 0.92	2.00 ± 0.77	0.82 ± 0.60	0.46 ± 0.52	80.09 ± 5.87	95.18 ± 1.40	96.46 ± 1.44
T-UKA	7.64 ± 0.92	5.70 ± 0.90	2.46 ± 0.82	0.27 ± 0.47	70.10 ± 5.87	93.73 ± 1.27	96.46 ± 1.21
*t* value	7.611	10.381	5.331	0.861	3.993	2.549	0.000
*p* value	<0.001	<0.001	<0.001	0.146	0.001	0.019	0.482

## Data Availability

The data supporting the findings is available in this manuscript, but we do not wish to share the data before it is published, because it is involved in the privacy of the patients, including age, sex, and the image of the radiology and intraoperative.

## Abbreviations

UKA: Unicompartmental knee arthroplasty
3DGT: Three-dimensional printed patient-customized guiding template
T-UKA: Traditional UKA
HKA: Hip–knee angle
HSS: Hospital for Special Surgery
OA: Osteoarthritis
MOA: Medial compartment knee osteoarthritis
TKA: Total knee arthroplasty
3D: Three-dimensional
CAD: Computer-aided design.
